# Extracorporeal life support in major trauma: case series from a tertiary referral trauma

**DOI:** 10.1186/cc10705

**Published:** 2012-03-20

**Authors:** R Spina, S Biondi, A Circelli, G Cianchi, S Degl'Innocenti, M Bonacchi, A Peris

**Affiliations:** 1 Careggi Teaching Hospital, Florence, Italy

## Introduction

Major trauma (MT) is a leading cause of death and disability worldwide. Immediate fatal complications are bleeding shock followed in the post-emergency phase by severe head and spine injury and post-traumatic respiratory failure. Venoarterial (va) or venovenous (vv) extracorporeal life support (ECLS) could represent a valid option to face these life-threatening trauma complications. Moreover, ECLS can potentially be employed to expand the pool of donors in patients with brain death diagnosis after trauma event.

## Methods

Patient data were collected from January 2009 to November 2011. A multidisciplinary algorithm-based protocol was written by our ECLS Team. The va-ECLS indications were bleeding shock, with potential controllable bleeding sites, not responding to massive fluid and blood resuscitation and to vasopressor support and cardiac arrest not responding after 10 minutes of cardiopulmonary resuscitation (CPR). vv-ECLS criteria establishment were severe hypoxia and/or hypercarbia due to acute lung failure not responding to conventional ventilatory strategies. The ECLS device is composed of a centrifugal pump and a hollow fiber membrane oxygenator (Quadrox-D; Maquet, Germany). Coagulation status was controlled by the activated partial thromboplastin time every 4 hours and thromboelastography.

## Results

A total of 19 trauma patients, 15 males and four females, underwent the ECLS technique. All of the following data are expressed as median and 25th and 75th percentiles are enclosed in parenthesis. Median age was 48 (31.8 to 63.8) years. Injury severity score was 59 (41.3 to 73.8). Thirteen patients had polytrauma with brain injury. Fourteen patients received va ECLS and five patients vv ECLS. Indications to ECLS placement were: cardiac arrest in nine patients, severe bleeding shock in five patients and acute respiratory failure in five patients. In four patients, ECLS was placed in the shock room, two patients received ECLS in the operating room during damage control surgery and 13 patients in the ICU. Timing to ECLS from the trauma event was within 6 hours for six patients, between 6 and 24 hours for five patients and over 24 hours for eight patients. Sixteen patients were admitted to the ICU. Five patients were discharged from the ICU. Brain death diagnosis as a consequence of traumatic injury was performed in six patients. In four of these patients organ donation was possible.

## Conclusion

ECLS in a multimodal approach in MT represents a challenging opportunity to face potential fatal complications. Moreover ECLS applied to selected trauma patients could expand the potential donor pool.

